# Why Brazil does not have an epidemic of chronic diseases: some answers from cardiovascular diseases

**DOI:** 10.1590/S1516-31802005000200001

**Published:** 2005-03-02

Nowadays, there is a notable dilemma for all societies: the increasing population of elderly people and society's difficulty in providing the necessary pension resources. The causes of such difficulties may be merely demographic or economic, such as reduced economic activity, more unemployment and informal work, with a consequent fall in pension fund revenue. In all societies in which a democratic social contract has been established, as is the case of Brazil, this problem may, to a greater or lesser extent, be demographically or economically determined. In the Brazilian case, it is very likely that the demographic question has less importance than does the socioeconomic question, since the greater proportion of elderly people stems from the reduced birth rate that has resulted from the abrupt fall in fertility rates, rather than from increased longevity due to medical care improvement. In consequence, the burden of non-transmissible chronic diseases is not a new issue, considering the risk of either disease or death, but only a manifestation of a higher proportion of elderly people.^1^

The term "non-transmissible chronic diseases" deserves comment, even though it is a practical term. It designates a set of different diseases, from the etiological point of view, as "non-infectious" and, from the temporal point of view, as "non-acute". Nevertheless, there are chronic diseases for which the basic cause is infectious transmittable agents, such as peptic ulcer (*Helicobacter pylori*) and carcinoma of the uterine neck (papillomavirus). A chronic course, however, can be observed for contagious infectious diseases such as Hansen's disease, malaria and paracoccidioidomycosis. The natural history of acute myocardial infarct and subarachnoid hemorrhage can be measured in minutes. For the purposes of this opening article, the best approach to public health management would be to abandon the wide range of chronic diseases to be analyzed in detail in their main groups, firstly by the chapters of the International Classification of Disease as cardiovascular diseases and cancers and then, secondly, by detailing the cerebrovascular and coronary diseases and the specific types of cancer. In the present text, we present the Brazilian panorama of cardiovascular diseases, in order to contest the idea of epidemics and the emergence of chronic diseases in the way that is suggested by texts that, in giving great prominence to the Asian example, make improper extrapolations of the Brazilian reality.^2^

As we will show in the following, using the example of cardiovascular diseases, the epidemic of cardiovascular diseases, or more appropriately, of cerebrovascular and coronary diseases, has already occurred and may even come back, considering the increases in obesity and diabetes.^3^ However, the real reason for this has never been the popularly ascribed reason of adoption of the Western lifestyle, or more exactly, the adoption of the American dietary patterns of the major fast-food chains such as McDonald's. This designation, as well as displaying a lack of knowledge of the planet's geography, has no empirical basis. It cannot, for example, be shown that "McDonaldization" has been adopted in the daily lives of the Brazilian population. On the contrary, there is evidence that the dietary modifications in the country are following their own course, which may even explain part of the obesity, albeit far from the American dietary patterns.^4^

The antiquity of the "cardiac disease" question in the country is shown in medical compendiums from the nineteenth century, such as "Lessons in Internal Medicine", which describes patients in Rio de Janeiro with angina and myocardial infarct, at a time when not only rheumatic cardiac disease but also syphilis was highly prevalent.^5^ The importance of chronic diseases in the profile of mortality in Brazil was established in the 1960s (i.e. 45 years ago), when the set of diseases of the circulatory system were superimposed on the infectious-parasitic diseases in the country's state capitals.^6^ However, the change to predominance of chronic diseases over infectious-parasitic diseases in the city of São Paulo took place immediately following the World War II (*circa* 1945-50). In 1900, among the ten main causes of deaths in São Paulo, there were "heart diseases" (third place, 6.5%) and "cerebral congestion and hemorrhage" (tenth, 2.1%). In 1930, among the ten most frequent causes, there were "heart diseases" (fourth, 4.8%), "other affections of the circulatory system" (sixth, 4.3%), "cancer" (seventh, 4.1%) and "cerebral congestion and hemorrhages" (ninth, 2.5%). On the other hand, in 1960, "heart diseases" were stated as the main cause (19.0%), followed in second place by "cancer" (12.1%) and then by "vascular lesions of the central nervous system" (7.7%). The tenth cause, with 2.1% was diabetes.^7^

A series of studies begun in São Paulo in 1937^8–13^ (Table 1) described the profile of cardiopathy in the first half of that century. Although those studies only recognized Chagas’ disease in the 1940s, the proportion of cases hospitalized with a diagnosis of hypertension and atherosclerosis was big enough to show the importance and relevance of these risk factors during that period. In 1948, a screening study for tuberculosis with workplace radiography examinations ("abreugrafia") on 39,488 workers in São Paulo found that 1.2% were cases of dilatated myocardiopathy.^14^

**Table 1:** Frequency of heart diseases in case series from studies performed and published in São Paulo between the 1930s and 1960s

Author, Year	Hypertension	Atherosclerosis	Rheumatic	Chagas	Syphilis
Author, Year	Hypertension	Atherosclerosis	Rheumatic	Chagas	Syphilis
Rubião-Meira^8^ 1937	10.5	19.0	12.0	–	21.0
Chiaverini^9^ 1940	31.1	19.3	21.1	–	19.7
Ramos^11^ 1949	67.6	5.5	18.5	–	2.6
Chiaverini^10^ 1948	31.0	22.0	16.0	–	13.0
Chiaverini^12^ 1951	69.3	8.7	15.9	–	1.6
Tranchesi^13^ 1951	23.4	20.7	23.7	10.9	7.4

Studies of mortality have shown that the evolution of cardiovascular diseases in the city of São Paulo in the 1940s to 1960s showed a decline in rheumatic heart disease and the emergence of ischemic and cerebrovascular disease. In 1940, rheumatic disease and coronary disease presented similar mortality rates. Thirty years later, deaths due to rheumatic disease corresponded to just 10% of those caused by coronary disease. The predominance of deaths due to coronary diseases, in relation to cerebrovascular diseases, was established in the city of São Paulo at the end of the 1940s.^15^

A comparative study by the Pan-American Health Organization on mortality among adults, carried out in São Paulo and Ribeirão Preto (and another ten cities) at the start of the 1960s already showed that the mortality rates due to diseases of the circulatory system among men in these two cities in the State of São Paulo were close to the values observed in the United Kingdom and the United States. However, Brazilian women had rates that were higher than for British and American women. For both sexes, the mortality associated with hypertension presented higher rates in these two cities of the State of São Paulo.^16^

In the middle of the 1980s, a fall in the mortality due to cerebrovascular and coronary disease was detected in the city of São Paulo^17–19^ and in the State of São Paulo.^20,21^ This tendency subsequently spread to all of the Brazilian metropolitan regions. In summary, the mortality due to coronary and cerebrovascular disease may still be high in comparison with rates in other countries,^22^ but these diseases present declining or even stable rates,^3^ and never increasing in the way that has been reported for regions in which there has been an improvement in the quality of the certification (Northeastern region) or radical alteration of the occupation of the space (Center-West region).^23^

It is important to emphasize that the declining trend in mortality preceded the introduction of more efficacious treatments for reducing the case-fatality of cerebrovascular and coronary disease. The hypothesis of expanding morbidity that postulates that the decline in mortality is due to the reduction in the case-fatality rates of diseases is extremely weak. The reduction in the mortality due to coronary disease began in the United States at the end of the 1960s, when the therapeutic arsenal was almost zero, in comparison with what there is today.^24^

In conclusion, there is no epidemic of chronic diseases or recent adoption of "Western habits" in Brazil.^25^ The increase in the proportion of the elderly population is the result of the fall in fertility rates^1^ and the impact of medical actions for increasing longevity are still imperceptible, given that the fall in mortality due to cardiovascular diseases preceded the adoption of new therapeutic methods. The difference between today and 50 years ago is the increase in the notion of citizenship and extension of benefits to all, especially those who are most affected by diseases. In this sense, there will be a greater medical care demand resulting from the pressure for screening and prevention measures, coming from those who previously were excluded from healthcare services.
